# Supplementary material for: Lack of Benefit of Extending Temozolomide Treatment in Patients with High Vascular Glioblastoma with Methylated MGMT
Source: Cancers (Basel). 2021 Oct 29;13(21):5420. doi: 10.3390/cancers13215420 (PMC8582449; doi:10.3390/cancers13215420)
Supplement: Supplementary file 1 [file cancers-13-05420-s001.zip › cancers-1393951-supplementary.pdf]

Supporting Information

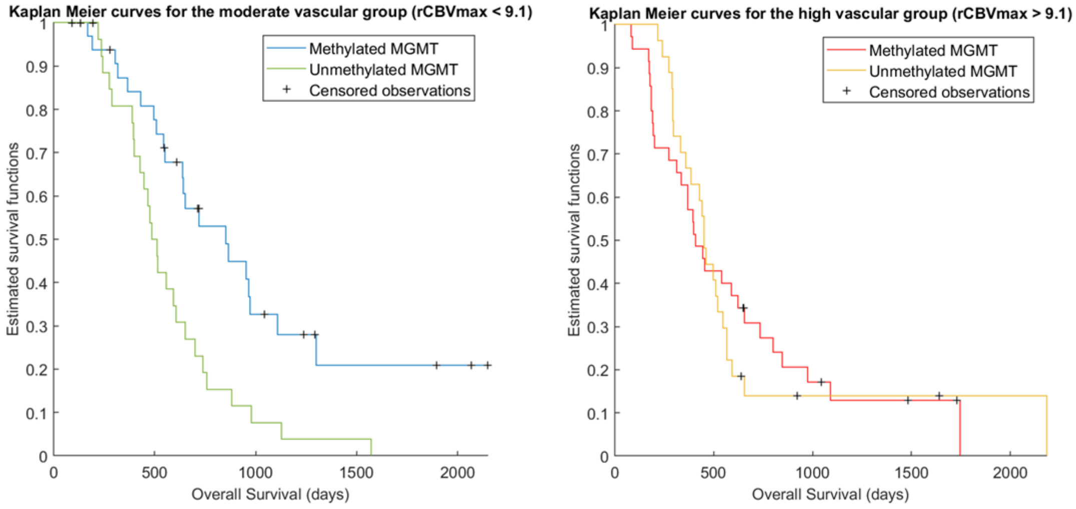

Figure S.1A: Kaplan Meier curves for the moderate vascular group (left) and for the high vascular group (right) depending on the *MGMT* methylation status. Vascular groups are defined by the threshold of the study cohort: median $rCBV_{max}$ (th = 9.1).

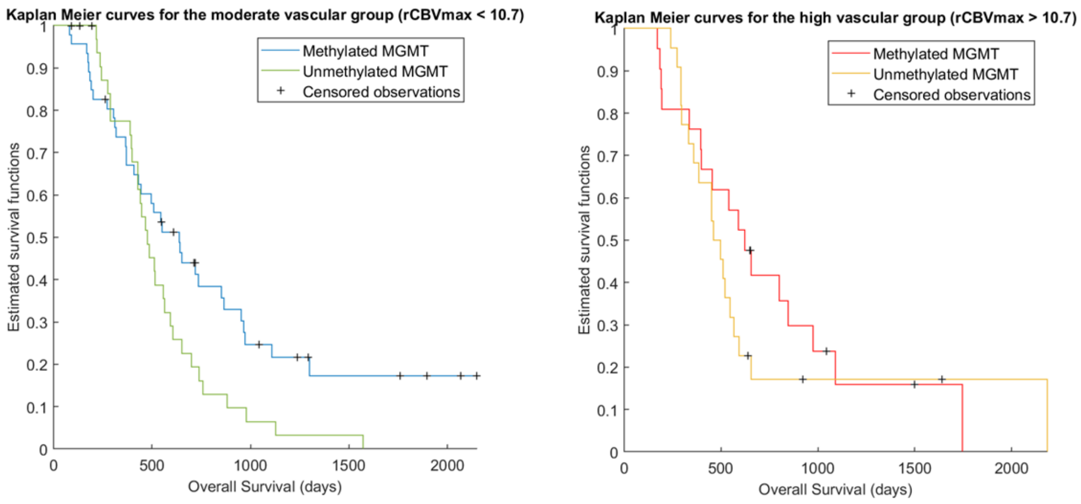

Figure S.1B: Kaplan Meier curves for the moderate vascular group (left) and for the high vascular group (right) depending on the *MGMT* methylation status. Vascular groups are defined by the threshold proposed in the previous study (49) (th = 10.7).
